# Identification and Analysis of the Mpp5Ab1-Interacting Protein in the Midgut of the *Colaphellus bowringi* Baly

**DOI:** 10.3390/toxins18060247

**Published:** 2026-05-29

**Authors:** Yaning Huang, Qiao Li, Jiaqi Wang, Yulei Wang, Daolong Liao, Xiaodong Sun, Haitao Li

**Affiliations:** 1 Key Laboratory of Vegetable Biology of Hainan Province, The Institute of Vegetables, Hainan Academy of Agricultural Sciences, Haikou 571100, China; hynamber@163.com (Y.H.); ldlshc@sina.com (D.L.); 2 College of Life Sciences, Northeast Agricultural University, Harbin 150030, China; liqiao020268@163.com (Q.L.); wangjiaqi1897@163.com (J.W.); wangyulei0622@163.com (Y.W.)

**Keywords:** *Bacillus thuringiensis*, *Colaphellus bowringi*, split-ubiquitin yeast two-hybrid, BiFC, molecular docking

## Abstract

To elucidate the mode of action of Mpp5Ab1 against *Colaphellus bowringi* Baly larvae, this study aimed to identify midgut proteins interacting with the toxin. A validated bait plasmid, pBT3-SUC-mpp5Ab1, was used to screen a larval midgut cDNA library via the split-ubiquitin yeast two-hybrid system. A total of 33 positive clones representing five distinct proteins were obtained, among which bioinformatic analyses prioritized three candidates: Cb-RP-L23e, Cb-CTSL, and Cb-TsetseEP. Subsequent bimolecular fluorescence complementation (BiFC) assays in Sf9 cells specifically confirmed interactions between Mpp5Ab1 and both Cb-CTSL and Cb-TsetseEP, whereas no fluorescence signal was observed for Cb-RP-L23e. Molecular docking further supported stable interactions between Mpp5Ab1 and the validated candidate proteins through hydrogen bonds, salt bridges, and hydrophobic interactions. These findings suggest that Cb-CTSL and Cb-TsetseEP may function as candidate interacting proteins associated with the activity of Mpp5Ab1 in the larval midgut of *C. bowringi*. Overall, this study provides new insight into the molecular interactions of Mpp5Ab1 and establishes a foundation for future functional studies on its insecticidal mechanism and receptor validation.

## 1. Introduction

*Bacillus thuringiensis* (Bt) is a Gram-positive bacterium within the *Bacillus* genus, which is widely distributed in soil ecosystems and is characterized by its production of insecticidal crystal proteins (ICPs) or δ-endotoxins during sporulation [1]. Most of these crystal proteins are acidic [2], remaining inert in the acidic gastric juice of vertebrates but dissolving and activating in the alkaline environment of the insect midgut. Subsequently, proteolytic cleavage yields active proteins of approximately 60 kDa, which target specific sites in insects and cause mortality. In addition to crystal proteins, during its vegetative growth phase, Bt can secrete vegetative insecticidal proteins (Vips) targeting Lepidoptera (Vip3) and Coleoptera (Vip1/Vip2). Another less-explored secreted protein, Mpp5Aa1 (formerly Sip1A), has also been described as an active agent against coleopteran pests [3].

The Sip protein was initially isolated from the culture supernatant of Bt strain EG2158 and named Sip1Aa1, now designated Mpp5Aa1. The mpp5Aa1 gene is 1104 bp long, encoding 367 amino acids with a molecular weight of approximately 41 kDa. The N-terminal 30 residues likely constitute a signal peptide cleaved during secretion to produce the mature protein of about 38 kDa [4]. Unlike Bt crystal proteins (Cry) and vegetative insecticidal proteins (Vips), the mode of action of Sip toxins is not fully elucidated. However, recent studies utilizing structural analysis and site-directed mutagenesis have shed light on the potential molecular basis of their insecticidal activity, providing important avenues for developing novel resistance management strategies. To date, two Sip proteins have been described in the literature, both of which are active against several coleopteran pests [5]. Strains harboring sip1Aa and sip1Ab genes also contain cry3 and cry8 genes, respectively, suggesting that Sip1 proteins may play a role in the insecticidal mechanism against Coleoptera [6]. Sip was the first and remains the only member of the family of Bt-secreted insecticidal proteins reported to be toxic to coleopteran larvae [6]; it shares homology with members of the ETX-Mtx2 family and is now classified within the Mpp family.

The Mpp family represents potential insecticidal proteins in Bt-containing metalloprotease domains that have functional characteristics distinct from classical Cry or Vip families [7]. Chen resolved the monomeric structure of Sip1Ab, revealing structural and sequential similarity to some ETX-MTX2 β-pore-forming toxins (β-PFTs). It possesses three domains and a unique amphipathic β-hairpin structure, highly similar to typical ETX-MTX2 proteins. Domain I is primarily responsible for receptor binding and varies significantly among different proteins; Domains II and III are involved in protein oligomerization and membrane pore formation. It is hypothesized that Mpp5Ab, similar to other ETX proteins, oligomerizes to form pores leading to membrane perforation. Mutations of the Sip1Ab residues Tyr118, His242, Asp290, and Ile307 significantly reduced insecticidal activity. Structural analysis indicated that three of these four residues are located in Domain I, suggesting that it may be the receptor-binding domain; as such, mutations in these three amino acids might hinder Sip1Ab binding to its receptor. The mutation of His242 in Domain III might impede the interaction between Sip1Ab and the host cell membrane [8].

*C. bowringi* is an oligophagous pest that primarily feeds on cruciferous vegetables, including Chinese cabbage, radish, and kale [9]. It is widely distributed across various provinces of China and in East and Southeast Asia. Both adults and larvae damage plants by gnawing on leaves, thus producing notches, holes, and skeletonization; severe infestations can lead to seedling loss and complete crop failure. The number of generations per year varies markedly with geography; for example, two have been reported in northern China, two to three in the Yangtze River Basin, and up to five or six in Guangxi. Adults exhibit a dual diapause strategy, entering both summer diapause (aestivation) and winter diapause (hibernation) in soil crevices or beneath leaf litter. Moreover, the pest’s hardened adult cuticle, death-feigning (thanatosis) behavior when disturbed, and pronounced generational overlap in the field collectively diminish the efficacy of chemical control and contribute to significant insecticide resistance. In recent years, the Bt insecticidal protein Mpp5Ab1 has shown considerable activity against this pest. Elucidating the molecular interaction mechanisms between Mpp5Ab1 and its midgut receptors, such as Cb-CTSL and Cb-TsetseEP, is expected to not only reveal a novel mode of action for this toxin but also provide a crucial theoretical foundation for developing highly targeted, environmentally friendly biopesticides and resistance management strategies.

Sha et al. successfully cloned the mpp5A gene from strain QZL38. This gene is 1095 bp long, encoding 364 amino acids with a molecular weight of approximately 41 kDa. Further functional validation showed that the Mpp protein maintained good stability under various conditions, including room temperature, UV exposure, trypsin digestion, and high temperature [10]. The insecticidal activity of the minimal active fragment was confirmed by removing the signal peptide. The truncated protein lacking the signal peptide (first 30 amino acids) exhibited insecticidal activity against *C. bowringi* Baly, with an LC_50_ of 1.078 μg mL^−1^, and showed no significant difference from the full-length protein [11]. In a similar vein, Shen et al. analyzed the expression of the Mpp5Ab1 protein in strain QZL38 and its heterologous expression in HD73. Using β-galactosidase assays, they analyzed the transcriptional activity of the Mpp5Ab1 gene promoter in HD73 wild-type and AbrB mutant strains. Secretion of the Sip1Ab1 protein in HD73 was nearly identical to that of the original QZL38 strain. Analysis of β-galactosidase activity in HD73 and AbrB mutant strains carrying Mpp5Ab1-lacZ indicated that sip1Ab1 gene transcription depends on AbrB, which can bind to the sip1Ab1 promoter. Two AbrB-binding sites were identified within the Mpp5Ab2 promoter region, demonstrating that AbrB positively regulates the expression of the Mpp5Ab1 gene [12]. Cao et al. investigated proteins interacting with the Mpp5Ab1 insecticidal protein in the brush border membrane vesicles (BBMVs) of *C. bowringi* Baly, and their competitive binding assay results indicated that Mpp5Ab has high binding affinity to *C. bowringi* Baly BBMVs [13]. In recent years, the Bt insecticidal protein Mpp5Ab1 has demonstrated significant activity against this pest. Investigating the molecular interactions between Mpp5Ab1 and midgut proteins like Cb-CTSL and Cb-TsetseEP may contribute to a better comprehension of the potential mechanism underlying its insecticidal activity. Identification of proteins associated with Mpp5Ab1 binding could offer valuable insights into how this toxin interacts with the larval midgut and may support future research aimed at clarifying its mode of action and developing environmentally friendly biopesticides and resistance management strategies.

## 2. Results

### 2.1. Yeast Two-Hybrid Screening for Mpp5Ab1-Interacting Proteins

Total RNA extracted from *C. bowringi* Baly larval midguts exhibited distinct 28S and 18S rRNA bands via spectrophotometry; the A260/A280 ratio of the RNA sample is 2.04.

The purity was high, confirming the sample’s suitability for library construction. The obtained double-stranded cDNA samples were analyzed using a spectrophotometer, yielding an A260/A280 value of 1.98. The RNA was similarly pure. The amplification of stably expressed reference genes ACT1 and RPL19 yielded bands of expected sizes, validating cDNA integrity for midgut cDNA library preparation.

The *C*. *bowringi* Baly midgut cDNA library bacterial stock, diluted 1000-fold, yielded 148 colonies on selective medium. The library titer was calculated as 2.96 × 10^7^ CFU/mL, with a total capacity of 1.48 × 10^8^ CFU, meeting standard construction criteria. Colony PCR of 23 randomly selected clones revealed insert sizes ranging from 400 to 3000 bp, averaging ~1000 bp, with >90% recombination efficiency. Thus, a yeast membrane system cDNA library, namely the larval midgut cDNA library of *C. bowringi* Baly, was successfully established, which is applicable to the screening of interacting proteins.

The mpp5Ab1 gene, devoid of its stop codon and N-terminal signal peptide, was amplified from the genomic DNA of *Bacillus thuringiensis* strain QZL38 and ligated into the pBT3-SUC vector to construct the recombinant bait plasmid pBT3-SUC-mpp5Ab1. Co-transformation of pBT3-SUC-mpp5Ab1 and the empty pPR3-N into NMY51 yeast cells led to growth on SD/-Leu/-Trp medium, verifying the successful transformation. No growth was detected on SD/-His/-Leu/-Trp or SD/-Ade/-His/-Leu/-Trp media, indicating that pBT3-SUC-mpp5Ab1 lacks autonomous transcriptional activation activity. Moreover, yeast transformed with pBT3-SUC-mpp5Ab1 or the empty pBT3-SUC vector showed similar growth on SD/-Trp medium, confirming that the bait plasmid is non-toxic to yeast. Functional validation using control strains demonstrated growth on all selective dropout media, confirming its compatibility with the split-ubiquitin system. Therefore, pBT3-SUC-mpp5Ab1 is non-toxic, autoactivation-negative, and appropriate for subsequent yeast two-hybrid library screening.

The hybridization solution was uniformly distributed on the SD/-Ade/-His/-Leu/-Trp quadruple dropout medium. Following an incubation period of 3–5 days, single colonies appeared, indicating successful interaction. Positive single colonies were chosen for secondary screening on the SD/-Trp/-Leu/His/-Ade/-X-α-gal dropout chromogenic medium, with NMY51[pTSU2-APP + pNubG-Fe65] and NMY51[pTSU2-APP + pPR3-N] serving as positive and negative controls, respectively. In total, 33 yeast-positive clones were isolated. These 33 positive clones were subjected to PCR amplification using the universal primers specific to the pPR3-N vector. The amplified products were then sequenced. After eliminating duplicate sequences and unsuccessful sequencing results, alignment analysis was carried out using BioEdit. As presented in Table 1, the results identified three distinct proteins. Based on sequencing quality and representativeness, these proteins were selected for subsequent experiments.

**Figure 1:**
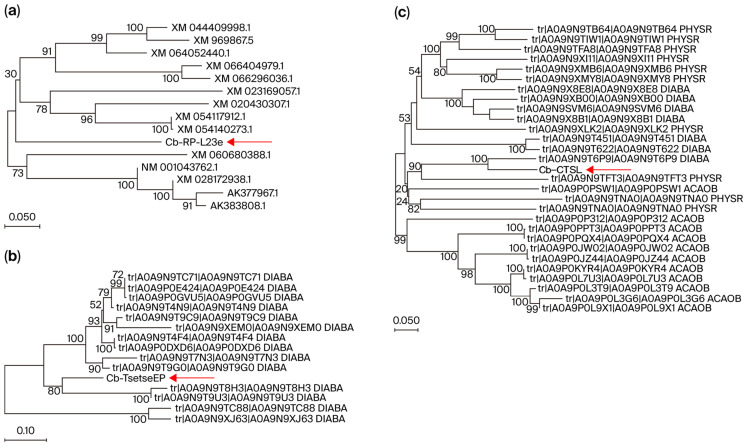
(**a**) Phylogenetic analysis of Cb-RP-L23e. (**b**) Phylogenetic analysis of Cb-CTSL. (**c**) Phylogenetic analysis of Cb-TsetseEP.

Multiple sequence alignment and phylogenetic tree construction were carried out for the candidate interacting proteins via the MEGAX (version 11) software. Homology analysis indicated that the three candidate proteins exhibited 100% homology with non-annotated proteins, and the genes with the highest homology and distinct functional annotations were ultimately chosen. They were named Cb-RP-L23e (displaying 99% homology with the 60S ribosomal protein L23 from Diabrotica virgifera), Cb-CTSL (showing 60% homology with Cathepsin L from Diabrotica speciosa), and Cb-TsetseEP (having 20.7% homology to a gut protein containing a Tsetse Threonine-rich Adhesin domain). The results of the phylogenetic tree demonstrated that Cb-RP-L23e shared over 80% similarity with ribosomal protein L23 proteins (Figure 1a), Cb-CTSL shared 41–61.9% homology with proteins from *Phyllotreta striolata* and *Diabrotica speciosa* (Figure 1c), and Cb-TsetseEP shared 19.1–48.6% homology with proteins from *Diabrotica speciosa* (Figure 1b).

**Figure 2:**
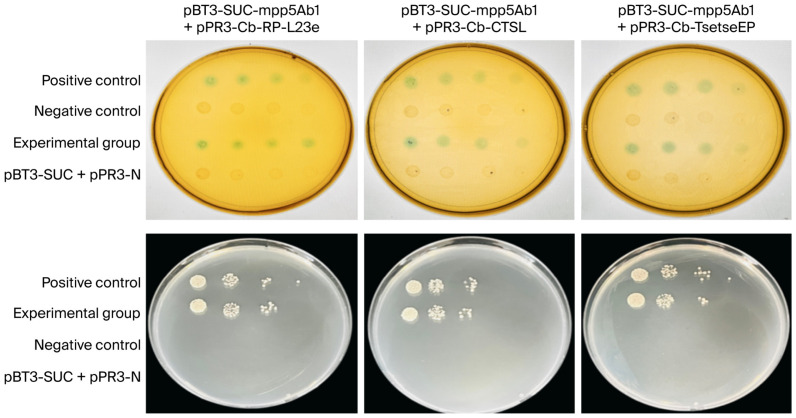
One-to-one backcross validation.

In subsequent yeast two-hybrid validation, prey vectors encoding candidate proteins were co-transformed with the bait vector pBT3-SUC-mpp5Ab1 into NMY51 yeast cells. Serial dilutions were spotted onto selective media alongside positive and negative controls. All three co-transformation groups grew on SD/-Ade/-His/-Leu/-Trp plates and exhibited blue coloration on SD/-Ade/-His/-Leu/-Trp/X-gal plates, confirming positive interactions between the candidate proteins and Mpp5Ab1 (Figure 2).

### 2.2. Molecular Docking of Mpp5Ab1 with Interacting Proteins

**Figure 3:**
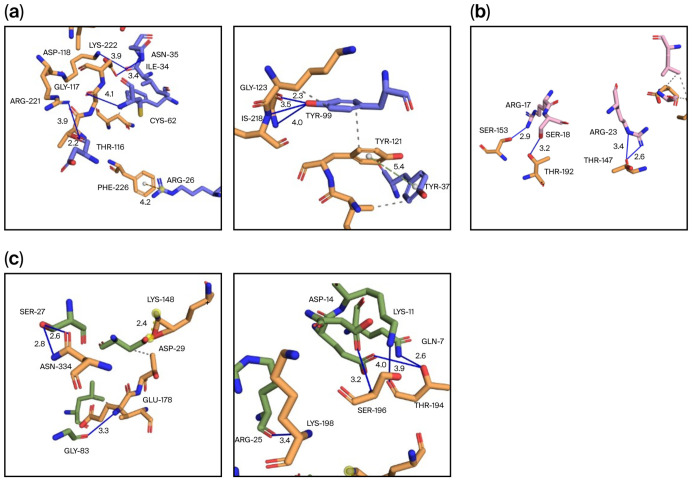
(**a**) Cb-RP-L23e-Mpp5Ab1 molecular docking. (**b**) Cb-CTSL-Mpp5Ab1 molecular docking. (**c**) Cb-TsetseEP-Mpp5Ab1 molecular docking.

Protein–protein docking between Mpp5Ab1 and Cb-RP-L23e, Cb-CTSL, and Cb-TsetseEP was performed using HDOCK and analyzed with PDBePISA, PLIP, and PyMol. For Cb-RP-L23e–Mpp5Ab1, the lowest-energy model yielded a docking score of −414.32, a confidence score of 0.9949, and a ΔiG of −10.4 kcal/mol, stabilized by eight hydrogen bonds, one π–cation interaction, and multiple hydrophobic contacts. As shown in Figure 3a, the yellow chain represents Mpp5Ab1, and the purple chain represents Cb-RP-L23e. A total of eight hydrogen bonds are formed between the two proteins. Specifically, a hydrogen bond (solid blue line) is formed between Lys-222 of Mpp5Ab1 and Ile-34 of Cb-RP-L23e; additional hydrogen bonds are formed between Asp-118 and Asn-35, between Arg-221 and Thr-116, and between Gly-117 and Cys-62. Furthermore, a π–cation interaction (orange dashed line) is formed between Phe-226 and Arg-26. As shown in panel B of the figure, hydrogen bonds are formed between Gly-123 and His-218 of Mpp5Ab1 and Tyr-99 of Cb-RP-L23e, and a π–π stacking interaction (green dashed line) is formed between Tyr-121 and Tyr-37. The Cb-CTSL–Mpp5Ab1 complex displayed a docking score of −620.93, a confidence score of 0.9999, and a ΔiG of −5.0 kcal/mol, involving four hydrogen bonds and hydrophobic interactions. As shown in Figure 3b, four hydrogen bonds are formed between Mpp5Ab1 (yellow chain) and Cb-CTSL (pink chain), including those between Ser-153 of Mpp5Ab1 and Arg-17 of Cb-CTSL, between Thr-192 and Ser-18, and between Thr-147 and Arg-23 (all indicated by solid blue lines). In addition, multiple hydrophobic interactions (gray dashed lines) are also present between the two proteins. For Cb-TsetseEP–Mpp5Ab1, the docking score was −643.26 with a confidence score of 0.9999 and a ΔiG of −8.8 kcal/mol, featuring eight hydrogen bonds, one salt bridge, and hydrophobic interactions. As shown in Figure 3c, eight hydrogen bonds and one salt bridge are formed between Mpp5Ab1 (yellow chain) and Cb-TsetseEP (green chain). Specifically, as shown in panel A of the figure, a hydrogen bond (solid blue line) is formed between Asn-334 of Mpp5Ab1 and Ser-27 of Cb-TsetseEP, a salt bridge (yellow dashed line) is formed between Lys-148 and Asp-29, and a hydrogen bond is formed between Glu-178 and Gly-83. As shown in panel B, a hydrogen bond is formed between Thr-194 of Mpp5Ab1 and Gln-7 of Cb-TsetseEP; hydrogen bonds are formed between Ser-196 and both Asp-14 and Lys-11, and a hydrogen bond is formed between Lys-198 and Arg-25. In addition, multiple hydrophobic interactions (gray dashed lines) are also present between the two proteins. These results indicate that all three candidate proteins exhibit a high binding probability with Mpp5Ab1 through diverse intermolecular interactions.

### 2.3. Expression of Mpp5Ab1-Interacting Proteins and In Vivo Validation by BiFC

**Figure 4:**
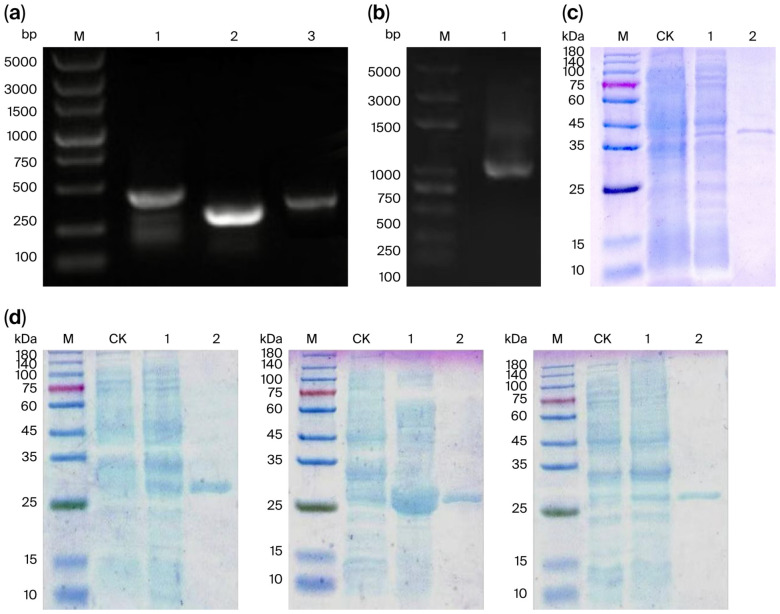
(**a**) Colony PCR verification of pull-down plasmid. M: DL5000 DNA marker; 1–3: pGEX-Cb-RP-L23e, pGEX-Cb-CTSL, pGEX-Cb-TsetseEP. (**b**) pET-28a-mpp5Ab1. (**c**) Purification of Mpp5Ab1-His protein. M: color-enhanced pre-stained protein molecular weight standards (10–180 kD); CK: pET-28a; 1: Mpp5Ab1-His crude protein; 2: Mpp5Ab1-His purified protein. (**d**) Purification of GST proteins. M: color-enhanced pre-stained protein molecular weight standards (10–180 kD); CK: pGEX-6P-1 empty vector; 1: crude purified target protein; 2: purified target protein.

To validate the interactions between the three potential interacting proteins (Cb-RP-L23e, Cb-CTSL, and Cb-TsetseEP) and Mpp5Ab1 in vitro, the study first constructed four recombinant plasmids: pGEX-Cb-RP-L23e, pGEX-Cb-CTSL, pGEX-Cb-TsetseEP, and pET-28a-mpp5Ab1. These were transformed into E. coli competent cells. Single colonies were picked for PCR verification using the corresponding universal primers. Electrophoresis on a 1.2% agarose gel showed bands of the expected sizes (Figure 4a,b), and sequencing confirmed no mutations, which verified successful recombinant plasmid construction. Target proteins were then purified. Mpp5Ab1-His, expressed from pET-28a-mpp5Ab1, was purified using a His-tag protein purification column, with the pET-28a empty vector as a negative control. SDS-PAGE showed expected bands of 37 kDa for both Mpp5Ab1-His crude and purified proteins (Figure 4c). Compared with the negative control, specific bands were observed for both the crude extracts and the purified proteins of GST-Cb-RP-L23e, GST-Cb-CTSL, and GST-Cb-TsetseEP. Because the target proteins were fused with a GST tag, the expected sizes of the expressed fusion proteins were 41 kDa, 36 kDa, and 40 kDa, respectively. The sizes observed via SDS-PAGE differed somewhat from the predicted sizes, which may be attributable to non-reducing tertiary structures or disulfide bonds.

**Figure 5:**
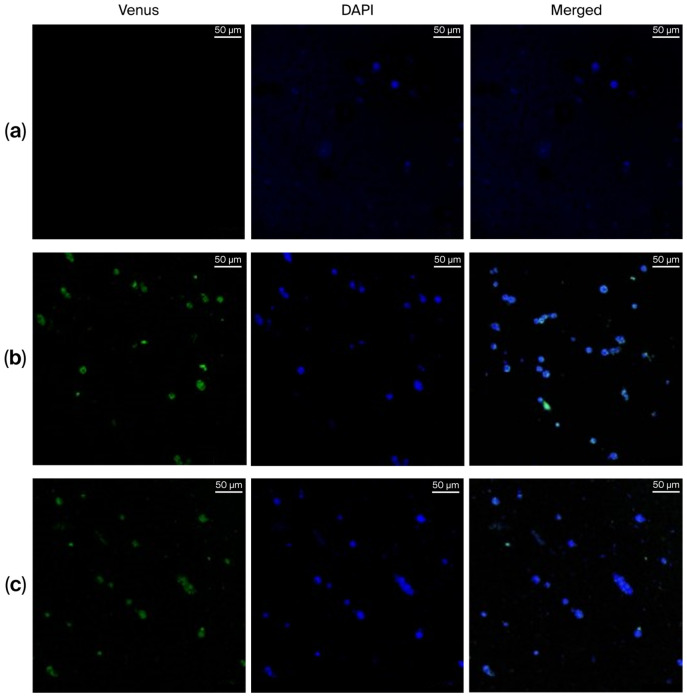
BiFC microscopic verification (**a**) BiFC-VC155-*mpp5Ab1* + BiFC-VN173-Cb-*RP-L23e.* (**b**) BiFC-VC155-*mpp5Ab1* + pBiFC-VN173-*Cb-CTSL.* (**c**) BiFC-VC155-*mpp5Ab1* + BiFC-VN173-*Cb-TsetseEP*.

To investigate the in vivo interaction of the screened potential interacting proteins, recombinant plasmids pBiFC-VC155-mpp5Ab1, pBiFC-VN173-Cb-RP-L23e, pBiFC-VN173-Cb-CTSL, and pBiFC-VN173-Cb-TsetseEP were constructed. The constructed plasmid vectors were co-transfected into Sf9 cells using jetPRIME^®^ transfection reagent. Specifically, pBiFC-VC155-mpp5Ab1 was co-transfected with each of the other three recombinant plasmids (pBiFC-VN173-Cb-RP-L23e, pBiFC-VN173-Cb-CTSL, and pBiFC-VN173-Cb-TsetseEP), resulting in three combinations. In principle, if proteins produced by the recombinant plasmids interact, fluorescence should be detected under a confocal laser scanning microscope, and, indeed, 48 h post-transfection, faint fluorescence signals were observed microscopically. Venus represents the fluorescent protein, DAPI stains the nuclei, and Merged is the overlay of the two channels. As shown in Figure 5, the combination of pBiFC-VC155-mpp5Ab1 with pBiFC-VN173-Cb-RP-L23e showed no fluorescence signal. In contrast, the combinations of pBiFC-VC155-mpp5Ab1 with pBiFC-VN173-Cb-CTSL and pBiFC-VN173-Cb-TsetseEP exhibited fluorescence signals under the microscope. This indicates that, in Sf9 cells, Mpp5Ab1 protein can interact with Cb-CTSL and Cb-TsetseEP proteins, bringing the N-terminal and C-terminal fragments of the Venus fluorescent protein into spatial proximity, thereby reconstituting fluorescence.

Additional figures related to the research findings can be found in the Supplementary Materials.

## 3. Discussion

The present study identifies specific midgut proteins from *C. bowringi* Baly that interact with the *Bacillus thuringiensis* insecticidal protein Mpp5Ab1, thereby offering critical insights into a previously uncharacterized mode of action. Classical Bt toxins, including Cry and Vip proteins [14], exert their effects primarily through well-established receptor-mediated pathways involving cadherin, aminopeptidase N (APN) [15], alkaline phosphatase (ALP), and ABC transporters [16].

In contrast, the Mpp protein family—distinguished by its metalloprotease domain architecture—has remained functionally enigmatic, with both its insecticidal mechanism and host interactors largely undefined. Our findings demonstrate that Mpp5Ab1 engages two proteins as binding partners in the larval midgut: Cb-CTSL, a lysosomal cathepsin L protease, and Cb-TsetseEP, a gut immunity-associated protein containing a TsetseEP domain. Notably, neither protein has been documented as a functional receptor for Cry or Vip toxins in Coleoptera, suggesting that Mpp5Ab1 operates through a distinct molecular pathway that challenges the prevailing receptor paradigm for Bt insecticidal proteins.

The interactions between Mpp5Ab1 and Cb-CTSL, as well as Cb-TsetseEP, verified via both split-ubiquitin yeast two-hybrid screening and BiFC assays, corroborate a multifaceted intoxication mechanism. Cb-TsetseEP might function as an initial docking site on the midgut epithelium, promoting toxin localization and potentially circumventing innate immune recognition, which is comparable to the anti-pathogen activities reported for tsetse fly homologs [17]. Additionally, TsetseEP proteins have been reported to contribute to safeguarding the insect midgut against pathogen colonization [18]. Hence, it is plausible that the interaction between Mpp5Ab1 and Cb-TsetseEP could be associated with physiological or immune-related processes transpiring in the larval midgut. However, whether this interaction entails immune modulation or other specific biological functions remains ambiguous and necessitates further experimental verification. Likewise, cathepsin L proteins are known to partake in lysosomal and physiological processes in insects [19]. Previous investigations have also demonstrated that cathepsins play crucial roles in insect development and digestion [20]. Moreover, digestive cathepsins have been identified in several coleopteran insects and are regarded as significant digestive proteases in these species [21]. Research on Plagiodera versicolora indicated the primary activity of Cathepsin L in the soluble fraction [22]. Thus, the observed interaction between Mpp5Ab1 and Cb-CTSL may potentially be linked to proteolytic processing or other midgut physiological activities. Nevertheless, the current study did not directly assess apoptosis, lysosomal membrane permeabilization, or toxin activation processes.

Recent studies have shown that ribosomal proteins (RPs) are not only involved in ribosome biogenesis but also possess extensive extra-ribosomal functions, participating in biological processes such as gene regulation, cell cycle, apoptosis, and DNA repair [23]. Although Cb-RP-L23e was repeatedly detected through yeast two-hybrid screening, subsequent BiFC validation failed to confirm a direct interaction between Cb-RP-L23e and Mpp5Ab1. This result suggests that the positive Cb-RP-L23e clone may be a false positive. Alternatively, the binding between Cb-RP-L23e and Mpp5Ab1 is extremely weak or requires additional auxiliary factors, thus preventing the formation of an intact fluorescent protein under BiFC conditions. Therefore, this study subsequently focuses on Cb-CTSL and Cb-TsetseEP, which were validated by both methods as direct interacting receptors of Mpp5Ab1.

The species-specific sequence divergence observed for Cb-CTSL and Cb-TsetseEP—both of which exhibit limited homology with their orthologs in other coleopteran pests—may provide a possible explanation for the differential activity spectrum of Mpp toxins. Furthermore, the intermolecular interactions predicted by molecular docking, including hydrogen bonds, salt bridges, and hydrophobic interactions, support the potential binding compatibility between Mpp5Ab1 and these candidate interacting proteins. From a methodological perspective, the successful application of the split-ubiquitin membrane yeast two-hybrid system in this study demonstrates its utility for identifying membrane-associated or secreted proteins that interact with Bt toxins.

Regarding the validation of protein interactions, while the bimolecular fluorescence complementation (BiFC) assays in Sf9 cells provide evidence for the direct binding between Mpp5Ab1 and both Cb-CTSL and Cb-TsetseEP, it is important to acknowledge a significant biological limitation. Sf9 cells are derived from the fall armyworm (Spodoptera frugiperda), a lepidopteran insect. Consequently, this heterologous expression system does not fully recapitulate the native cellular environment of the coleopteran host, *C. bowringi*. Factors such as membrane lipid composition, the presence of specific auxiliary proteins, and post-translational modification pathways can differ substantially between lepidopteran and coleopteran cells. Therefore, while the Sf9-based BiFC system is a well-established and valuable tool for confirming direct protein -protein interactions, a positive result in this system does not unequivocally prove that the same interaction is required for toxicity or occurs in the same manner within the *C. bowringi* midgut.

From a broader methodological perspective, the successful application of the split-ubiquitin membrane yeast two-hybrid system in this study demonstrates its utility for capturing membrane-proximal or secreted protein interactions that are refractory to conventional nuclear yeast two-hybrid approaches. However, some limitations should be noted. First, we have not established whether these interactions are required for insecticidal activity; functional validation (e.g., RNAi or CRISPR knockout) is needed to confirm them as true functional receptors. Quantitative binding assays and mechanistic hypotheses (apoptosis, immune modulation) also await future investigation. Although fluorescence signals were observed in the BiFC assays for the Cb-CTSL and Cb-TsetseEP combinations, the present analysis was primarily qualitative. Quantitative fluorescence analysis and additional biochemical validation will be necessary in future studies to further evaluate the strength and specificity of these interactions. Despite these limitations, our findings provide a molecular foundation for understanding the action of Mpp5Ab1 and developing receptor-based resistance management strategies.

## 4. Conclusions

This study identified three candidate proteins—CbRP-L23e, CbCTSL, and CbTsetseEP—that interact with the insecticidal protein Mpp5Ab1 in the larval midgut of *C. bowringi* Baly using the split-ubiquitin yeast two-hybrid system. BiFC assays and molecular docking analyses confirmed that CbCTSL and CbTsetseEP directly associate with Mpp5Ab1 via multiple intermolecular forces, whereas CbRP-L23e was not validated as a true binding partner. These results present the first experimental evidence that Mpp5Ab1 targets unique midgut proteins distinct from conventional Bt toxin receptors, offering novel insights into its mode of action. Furthermore, this work demonstrates the effectiveness of the split-ubiquitin membrane yeast two-hybrid system for discovering protein interactors of secreted Bt toxins, establishing a methodological basis for future receptor validation and resistance management studies against coleopteran pests.

## 5. Materials and Methods

### 5.1. Media, Bacterial Strains, Cell Line, and Insects

The YPDA medium, yeast-deficient medium, and YPD plus liquid medium used in this study were all procured from Beijing Coolaber Technology Co., Ltd (Beijing, China). Strain QZL38 was previously isolated and maintained in our laboratory to generate Mpp5Ab1. Sf9 (Spodoptera frugiperda) cells and complete medium were purchased from Wuhan Procell Life Science Technology Co., Ltd. (Wuhan, China). The complete medium consisted of TNM-FH medium supplemented with 10% (*v*/*v*) fetal bovine serum and 1% penicillin–streptomycin. Cells were cultured statically in air at 26–28 °C in a constant-temperature cell incubator (Yiheng Scientific Instruments Co., Ltd., Shanghai, China) and were routinely passaged at a split ratio of 1:3 to 1:5 every 3–4 days when reaching 80–90% confluence. Ovum samples of the leaf-mining moth were kindly provided by the Biotechnology Research Laboratory, Institute of Plant Protection, Chinese Academy of Agricultural Sciences (Beijing, China). The ovum feeding process was conducted in an intelligent climate-controlled chamber maintained at a constant temperature of 25 °C. The photoperiod was set to L12:D12, with relative humidity maintained between 40% and 60%.

### 5.2. Protein Expression and Purification

To express the Mpp5Ab1 protein, the mpp5Ab1 gene was cloned into the pET-28a(+) vector using oligonucleotide primers mpp5Ab1-F and mpp5Ab1-R for vector construction. To express the identified interacting proteins Cb-RP-L23e, Cb-CTSL, and Cb-TsetseEP, the genes were amplified using primer pairs Cb-RP-L23e-F/Cb-RP-L23e-R, Cb-CTSL-F/Cb-CTSL-R, and Cb-TsetseEP-F/Cb-TsetseEP-R, respectively. The vectors were digested with XhoI and BamHI, and the gene fragments were ligated into the pPR3-N and pGEX-6P-1 vectors (Novagen, Nanjing Novozan Biotechnology Co., Ltd. Nanjing, China) using T4 DNA ligase for vector construction. Successfully constructed plasmids were transformed into *E. coli* BL21 (DE3) (Invitrogen, Beijing Coolaber Technology Co., Ltd. Beijing, China) for expression and purification. Induction was carried out at 16 °C overnight with IPTG added at a 1:1000 (*v*/*v*) dilution in the liquid culture medium. His-tagged proteins were purified using cOmplete His-Tag Purification Resin (Roche, BBI Life Sciences Limited., Shanghai, China), and GST-tagged proteins were purified using GST-sepharose affinity columns (GE Healthcare, Abmart Medical Technology Co., Ltd. Shanghai, China). After ultrasonic lysis, the supernatant was incubated with Ni-NTA resin in binding buffer (50 mM Tris-HCl, pH 7.5, 300 mM NaCl, 10 mM imidazole) at 4 °C for 1 h. The resin was washed sequentially with wash buffer (50 mM Tris-HCl, pH 7.5, 300 mM NaCl) containing 20 mM and 50 mM imidazole, and the target protein was eluted with elution buffer (50 mM Tris-HCl, pH 7.5, 300 mM NaCl, 250 mM imidazole). The purity of the purified protein was assessed using 12% SDS-PAGE followed by Coomassie Blue staining, revealing a single band with >95% purity.

All strains and plasmids used in this study are listed in the Supplementary Materials.

### 5.3. RNA Extraction and cDNA Library Construction

RNA was extracted and purified from *C. bowringi* Baly larval midgut tissue using FreeZol Reagent, Dilution Buffer, and the PureLink RNA Mini Kit. Midgut tissue of *C*. *bowringi* larvae stored at −80 °C was collected and homogenized in FreeZol Reagent on ice. After chloroform extraction, the aqueous phase was collected and treated with Dilution Buffer. The sample was then precipitated with 70% ethanol (prepared with DEPC-treated water) and loaded onto a PureLink RNA Mini Kit purification column. Following the washing steps, the RNA was eluted with DEPC-treated water. The quality of the extracted RNA was assessed via agarose gel electrophoresis and the OD260/280 ratio, which was 2.04. The cDNA library was constructed using the SMART cDNA Library Construction Kit.

### 5.4. Yeast Two-Hybrid Library Screening

The split-ubiquitin membrane yeast two-hybrid (MYTH) system is specifically designed to study protein–protein interactions involving membrane proteins. This system is based on split-ubiquitin technology, in which the N-terminal half (Nub) and C-terminal half (Cub) of ubiquitin are fused to the bait and prey proteins, respectively. The bait protein is fused to the Cub moiety along with an artificial transcription factor (TF). The prey protein is fused to a mutated Nub (NubG) that has low affinity for Cub. When the bait and prey proteins interact, NubG and Cub are brought into proximity to reconstitute a functional ubiquitin molecule. The reconstituted ubiquitin is then recognized by ubiquitin-specific proteases (UBPs), which cleave the linker, releasing the transcription factor. The released transcription factor translocates to the nucleus and activates reporter gene expression (HIS3, ADE2, and lacZ), enabling the detection of positive interactions.

First, the validated bait strain was activated and expanded. Cell density was monitored by OD_600_, and NMY51 yeast-competent cells were prepared through washing and treatment with LiOAc/TE master mix. Second, a transformation system containing the library plasmid, PEG/LiOAc master mix, denatured carrier DNA, and competent cells was set up. After water bath incubation, heat shock, and recovery, the bait yeast culture was mated with the library culture. Successful mating was confirmed via microscopic observation of cloverleaf-shaped zygotes. Finally, the mating mixture was plated on SD/-Ade/-His/-Leu/-Trp quadruple dropout medium for primary screening. Positive single colonies were picked and, along with positive and negative control sets, were spotted onto quadruple dropout selection medium containing X-α-gal for secondary screening. Ultimately, blue positive clones were selected for back-validation, thereby completing the screening of target proteins. A total of 33 positive clones were screened.

### 5.5. Phylogenetic Tree Construction

The obtained candidate positive clones were identified using PCR. The resulting sequencing data were aligned with the *C. bowringi* Baly larval midgut transcriptome using BioEdit (version 4.7.6), followed by BLAST (version 2.12.0) searches against the NCBI blastx online tool (https://blast.ncbi.nlm.nih.gov/Blast.cgi, accessed on 6 May 2024), the InsectBase database (http://www.insect-genome.com/, accessed on 6 May 2024), and the UniProt BLAST database (https://www.uniprot.org/blast/, accessed on 7 May 2024) for homology analysis and phylogenetic tree construction.

### 5.6. Molecular Docking

Protein structures were predicted using I-TASSER (version 2.12.0) (https://zhanggroup.org/I-TASSER-MTD/, accessed on 7 May 2024). HDOCK (V 1.1) was used to predict the most likely three-dimensional conformation of two proteins upon binding. The PDBePISA (version 2.0) server was used to calculate the model’s ΔiG. The most probable 3D models were analyzed using the PLIP (V2.2.2) interaction analysis platform, and the results were visualized using PyMol (version 2.5.x). The results showed that the lowest-energy model yielded a docking score of −414.32, a confidence score of 0.9949, and a ΔiG of −10.4 kcal/mol calculated using the PDBePISA server.

### 5.7. Plasmid Construction, Transfection, and In Vivo Validation

Restriction endonucleases EcoRI and SalI were used to linearize the plasmids BiFC-VC155 and BiFC-VN173. The target genes were cloned into the linearized plasmids to create expression vectors, denoted as BiFC-VC155-mpp5Ab1, BiFC-VC173-Cb-RP-L23e, BiFC-VC173-Cb-CTSL, and BiFC-VC173-Cb-TsetseEP. Cells were transfected using jetPRIME^®^ transfection reagent. Each of the following combinations was transfected: 2 μg BiFC-VC155-mpp5Ab1 plasmid with 2 μg BiFC-VC173-Cb-RP-L23e, BiFC-VC173-Cb-CTSL, and BiFC-VC173-Cb-TsetseEP. Each plasmid group was transfected in triplicate. Following transfection, cells were fixed with 4% PFA at room temperature for 10 min. Cells were then stained with 500 µL of 1 ng/µL DAPI nuclear stain at room temperature in the dark for 5 min. After staining, fluorescent signals were observed under a confocal laser scanning microscope. The confocal parameters included Venus (excitation 514 nm, emission 520–550 nm) and DAPI (excitation 405 nm, emission 410–480 nm) Settings; use of a 63× oil-immersion objective; and constant laser power, pinhole, gain, and offset across all groups. Additional settings included laser power set to 1–2%, pinhole set to 1 Airy unit, and photomultiplier tube (PMT) gain and offset kept consistent across all samples to ensure comparability of fluorescence intensity.

## Figures and Tables

**Table 1:** Mpp5Ab1-interacting proteins screened via the split-ubiquitin MYTH system.

Gene ID/Name	Function	Pathway
XM_050648012.1	RP-L23e, RPL23; large subunit ribosomal protein L23e	Ribosome
CEUTPL_LOCUS5678	CTSL; cathepsin L [EC:3.4.22.15]	Lysosome; Apoptosis
DIABBA_LOCUS11452	Protein TsetseEP domain-containing protein	Gut Protein

## Data Availability

The original contributions presented in this study are included in the article/Supplementary Material. Further inquiries can be directed to the corresponding authors.

## Supplementary Materials

The following supporting information can be downloaded at: https://www.mdpi.com/article/10.3390/toxins18060247/s1, Figure S1: Electrophoretic of midgut RNA of Colaphellus bowringi Baly; Figure S2: Electrophoretic analysis of PCR for mpp5Ab1 geneM: DL2000 Marker, 1: mpp5Ab1 gene; Figure S3: Electropherogram of reference gene. M: DL5000 DNA marker, 1: RPL19 gene, 2: ACT1 gene; Figure S4: Construction of cDNA Library of the Large Rhinoceros Beetle. A,Determination of cDNA Library Titer. B,Colony PCR Electrophoresis of Insert Fragmen; Figure S5: (A) Self-activation test of the bait plasmid. (B) Toxicity Assessment of Recombinant Plasmid. (C) Functional verification test of the bait plasmid; Figure S6: Microscopic Examination of Hybridization Solutio; Figure S7: Colonies on QDO plate; Figure S8: Screening resµLts of interacting proteins; Figure S9: Colony PCR Verification of BiFC Plasmid. M: DL5000 DNA marker, 1-3: Reconstituted coloniesPCR. Table S1: The strains and plasmids used in this study; Table S2: The primary restriction endonucleases and biochemical reagents used in this study; Table S3: The preparation methods for the primary solutions and buffers used in this study.
