# Supplementary material for: Identification and Analysis of the Mpp5Ab1-Interacting Protein in the Midgut of the Colaphellus bowringi Baly
Source: Toxins (Basel). 2026 May 29;18(6):247. doi: 10.3390/toxins18060247 (PMC13308108; doi:10.3390/toxins18060247)
Supplement: Supplementary file 1 [file toxins-18-00247-s001.zip › toxins-4236347-Supplementary.pdf]

Supplementary Materials: Identification and Analysis of the Mpp5Ab1-Interacting Protein in the Midgut of the *Colaphellus bowringi* Baly

Yaning Huang, Qiao Li, Jiaqi Wang, Yulei Wang, Daolong Liao, Xiaodong Sun and Haitao Li

Table S1. The strains and plasmids used in this study.

Strains & plasmids	Genotype & Characterization
Strains	
QZL38	*mpp5Ab1* gene
NMY51[pTSU2-APP + pPR3-N]	positive control
NMY51[pTSU2-APP + pNubG-Fe65]	negative control
Chemically Competent Cell	
DH5α	F-, recA1, deoR, endA1, phoA, hsdR17 (rk-mk +), λ-, supE44, thi-1, relA1, gyrA96, Δ (lacZYA-argF)U169
NMY51	MATa, trp1-901, leu2-3, 112, ade2, GAL4, his3Δ200, ura3:: (lexAop)8-lacZ, LYS2:: (lexAop)4-HIS3, ade2:: (lexAop) 8-ADE2
TOP10	F-, △lacX74, mcrAΔ (mrr-hsdRMS-mcrBC), lacZΔM15, φ80, araΔ139Δ (ara-leu), 7697recA1, galU/galK, (StrR) endA1, rps, nupG
BL21 (DE3)	ompT, F-, gal, hsdSB (rB- mB-), dcm (DE3),
Plasmids	
pBT3-SUC	T7 promoter, 7624 bp, Kan^r
pPR3-N	CYC1 promoter, 6200 bp, Amp^r
pET-28a	T7/lac promoter, 5633 bp, Kan^r
pGEX-6P-1	Tac promoter, 4984 bp, Amp^r
pBT3-SUC-*mpp5Ab1*	pBT3-SUC plasmid harboring *mpp5Ab1* gene, Kan^r
pPR3-N-*Cb-RP-L23e*	pPR3-N plasmid harboring the *Cb-RP-L23e* gene, Amp^r
pPR3-N-*Cb-CTSL*	pPR3-N plasmid harboring the Cb-*CTSL* gene, Amp^r
pPR3-N-*Cb-TsetseEP*	pPR3-N plasmid harboring the Cb-*TsetseEP* gene, Amp^r
pET-28a-*mpp5Ab1*	pET-28a plasmid harboring the *mpp5Ab1* gene, Kan^r
pGEX-*Cb-TsetseEP*	pGEX-6P-1 plasmid harboring *Cb-TsetseEP* gene,Amp^r
pGEX-*Cb-RP-L23e*	pGEX-6P-1 plasmid harboring *Cb-RP-L23e* gene,Amp^r
pGEX- *Cb-CTSL*	pGEX-6P-1 plasmid harboring *Cb-CTSL* gene,Amp^r
BiFC-VC155-*mpp5Ab1*	Venus plasmid harboring *mpp5Ab1* gene,Amp^r
BiFC-VN173-*Cb-RP-L23e*	Venus plasmid harboring *Cb-RP-L23e* gene,Amp^r
BiFC-VN173-*Cb-TsetseEP*	Venus plasmid harboring *Cb-TsetseEP* gene,Amp^r
BiFC-VN173-*Cb-CTSL*	Venus plasmid harboring *Cb-CTSL* gene,Amp^r

Table S2. The primary restriction endonucleases and biochemical reagents used in this study.

Restriction endonuclease and biochemical reagent	Company	Country
2 × Phanta® Max Master Mix	Nanjing Novazeen Biotechnology Co., Ltd.	CHN
6×DNA Sample loading buffer	Biyun Tian Biotechnology Research Institute	CHN
DL5000 DNA Marker	Nanjing Novazeen Biotechnology Co., Ltd	CHN
2 × Taq Master Mix (Dye Plus)	Nanjing Novazeen Biotechnology Co., Ltd	CHN
Color-Enhanced Pre-Stained Protein Molecular Weight Standards（10-180 kD）	Shandong Sparkjade Biotechnology Co., Ltd.	CHN
5 × SDS-PAGE Loading Buffer	Jiangsu Kangwei Century Biotechnology Co., Ltd.	CHN
SMART® cDNA Library Construction Kit	Baobi Biotechnology Co., Ltd.	JPN
3-Amino-1,2,4-triazole (3-AT)	Beijing Zhuangmeng International Bio-Genetic Technology Co., Ltd.	CHN
SfiI	NEB Biotechnology (Beijing) Co., Ltd.	CHN
BamHI	NEB Biotechnology (Beijing) Co., Ltd.	CHN
XhoI	NEB Biotechnology (Beijing) Co., Ltd.	CHN
EcoI	NEB Biotechnology (Beijing) Co., Ltd.	CHN
SalI	NEB Biotechnology (Beijing) Co., Ltd.	CHN
ClonExpress II One Step Cloning Kit	Nanjing Novazeen Biotechnology Co., Ltd.	CHN
Rapid Plasmid DNA Mini Prep Kit	Hangzhou Xinjing Biological Reagent Development Co., Ltd.	CHN
Gel DNA Recovery Kit	Hangzhou Xinjing Biological Reagent Development Co., Ltd.	CHN
FreeZol Reagent	Nanjing Novazeen Biotechnology Co., Ltd.	CHN
PureLink RNA Mini Kit	Thermo Fisher Scientific Technologies Co., Ltd.	USA
RNA Enzyme and Nucleic Acid Removers	Nanjing Novazeen Biotechnology Co., Ltd.	CHN
Native-PAGE Rapid Preparation Kit for Non-Denaturing Acrylamide Gels	BBI Life Sciences Co., Ltd.	CHN
5 × Native Sample Loading Buffer	Shenggong Bioengineering (Shanghai) Co., Ltd.	CHN
QuickBlock™ Western Sealing fluid	Biyuntian Biotechnology Research Institute	CHN

His-tag Antibody (mouse monoclonal antibody)	Biyuntian Biotechnology Research Institute	CHN
BeyoECL Plus	Biyuntian Biotechnology Research Institute	CHN
HyPur T Ni-NTA 6FF (His-Tag) Pre-loaded Gravity Column Purification Kit	BBI Life Sciences Co., Ltd.	CHN
Serum-Free Non-Programmed Freezing Medium	Wuhan Punoise Life Technology Co., Ltd.	CHN
Horseradish peroxidase-labeled goat anti-mouse IgG(H + L)	Biyuntian Biotechnology Research Institute	CHN
12%SDS-PAGE Rapid Color Gel Preparation Kit	Shandong Sparkjade Biotechnology Co., Ltd.	CHN
jetPRIME® transfection reagent	Polyplus Transferion® Society Anonymous	FRA

Table S3. The preparation methods for the primary solutions and buffers used in this study.

Solution and buffer	Preparation
1 M Tris-HCl (pH6.8)	12.1g Tris base dissolved in 80 mL deionized water, adjusted to pH 6.8 with approximately 7.2 mL concentrated HCl, diluted to 100mL with deionized water, autoclaved at 120°C for 20 minutes, stored at room temperature
1 M Tris-HCl (pH8.8)	Dissolve 12.1g of Tris base in 80mL of deionized water. Adjust the pH to 8.8 with approximately 4mL of concentrated HCl. Dilute to 100mL with deionized water. Autoclave at 120°C for 20 minutes. Store at room temperature.
LiOAc/TE master mix	1M LiOAc 1.1mL, 10 × TE (pH7.5) 1.1mL, ddH_2O 7.8mL
PEG/LiOAc master mix	50%PEG 12mL, 1 M LiOAc 1.5mL, 10 × TE (pH7.5) 1.5mL
Combined Buffer Solution(1L)	1M LiOAc 1.1mL, 20mM $NaH_2PO4 \cdot 2H_2O$ 3.1202g, 20Mm $Na_2HPO4 \cdot 7H_2O$ 5.3614g, 0.5M NaCl 29.22g
Wash Buffer(1L)	20mM $NaH_2PO4 \cdot 2H_2O$ 3.1202 g, 20 Mm $Na_2HPO4 \cdot 7H_2O$ 5.3614g, 0.5 M NaCl 29.22g,1~30mM Imidazole 0.06808g~2.0424g
Elution Buffer(1L)	20mM $NaH_2PO4 \cdot 2H_2O$ 3.1202 g, 20 Mm $Na_2HPO4 \cdot 7H_2O$ 5.3614g, 0.5M NaCl 29.22g, 500mM Imidazole 34.04g
10%AP(Ammonium persulfate)	Dissolve 0.1g of AP powder in 1 mL of sterile water. Prepare immediately before use and store at 4°C for no longer than two weeks.
10%SDS	Dissolve 10g SDS in 90mL deionized water, adjust to 100mL, aliquot, and store at -20°C.
SI (500mL)	200mL distilled water, 250mL anhydrous ethanol, 50mL glacial acetic acid
SIII (100mL)	0.25g Coomassie Brilliant Blue R250 dissolved in 100mL anhydrous ethanol

3 M Sodium acetate (pH5.2)	40.8g $NaAc{\cdot}3H_2O$ added to 40mL deionized water, adjusted to pH5.2 with glacial acetic acid, diluted to 100mL with deionized water, autoclaved, and stored at room temperature
Electrophoresis Buffer (1L)	Tris base 3.02g, Gly 18.8g, SDS 1g dissolved in 1L deionized water
EtBr	1.0g EtBr dissolved in 100mL deionized water; store in a brown bottle at room temperature protected from light
20% glycogen	Dissolve 20g maltose in 80mL deionized water, adjust to 100 mL, filter sterilize, store at 4°C
Non-denaturing electrophoresis buffer (1L)	Tris base 3.02g, Gly18.8g dissolved in 1L deionized water
Transfer Buffer (1L)	Tris base 3.03g, Gly 14.4 g, 20%methanol
x-α-Gal (20 mg/mL)	20mg X-α-Gal dissolved in 1mL DMF, sterilized by filtration through a 0.22μm organic nylon membrane, stored at -20°C protected from light
1 M IPTG	2g IPTG dissolved in 8mL sterile water, sterilized by filtration through a 0.22 μm organic nylon membrane, stored at -20°C
1 × TBS (1L)	20mL 1MTris-HCl (pH7.5-7.6), 8g NaCl stored at room temperature
TBST	1×TBS supplemented with 0.2% Tween20
0.9%NaCl	0.9g NaCl dissolved in 100mL deionized water
0.1M $CaCl_2$	5.5g $CaCl_2$ dissolved in 500mL deionized water, filtered through a 0.22μm inorganic filter membrane

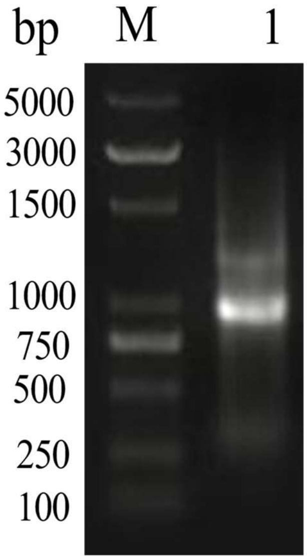

Figure S1. Electrophoretic of midgut RNA of Colaphellus bowringi Baly.

Notes： M: DL5000 DNA marker, 1: RNA

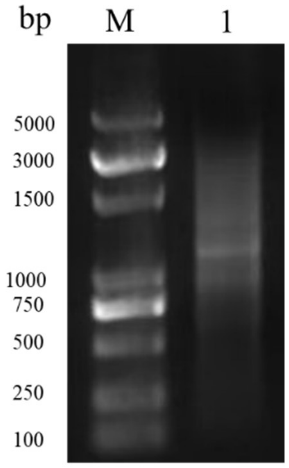

Figure S2. Electrophoretic analysis of PCR for mpp5Ab1 geneM: DL2000 Marker, 1: *mpp5Ab1* gene.

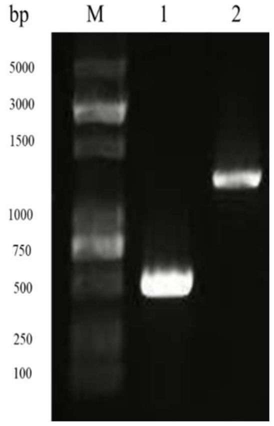

Figure S3. Electropherogram of reference gene. M: DL5000 DNA marker, 1: RPL19 gene, 2: ACT1 gene.

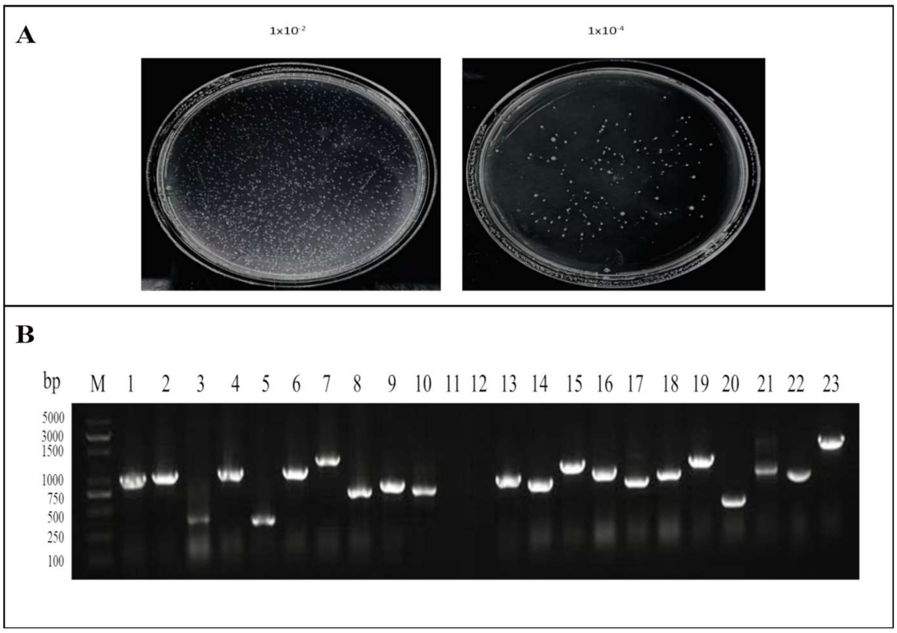

Figure S4. Construction of cDNA Library of the Large Rhinoceros Beetle. A, Determination of cDNA Library Titer. B, Colony PCR Electrophoresis of Insert Fragmen.

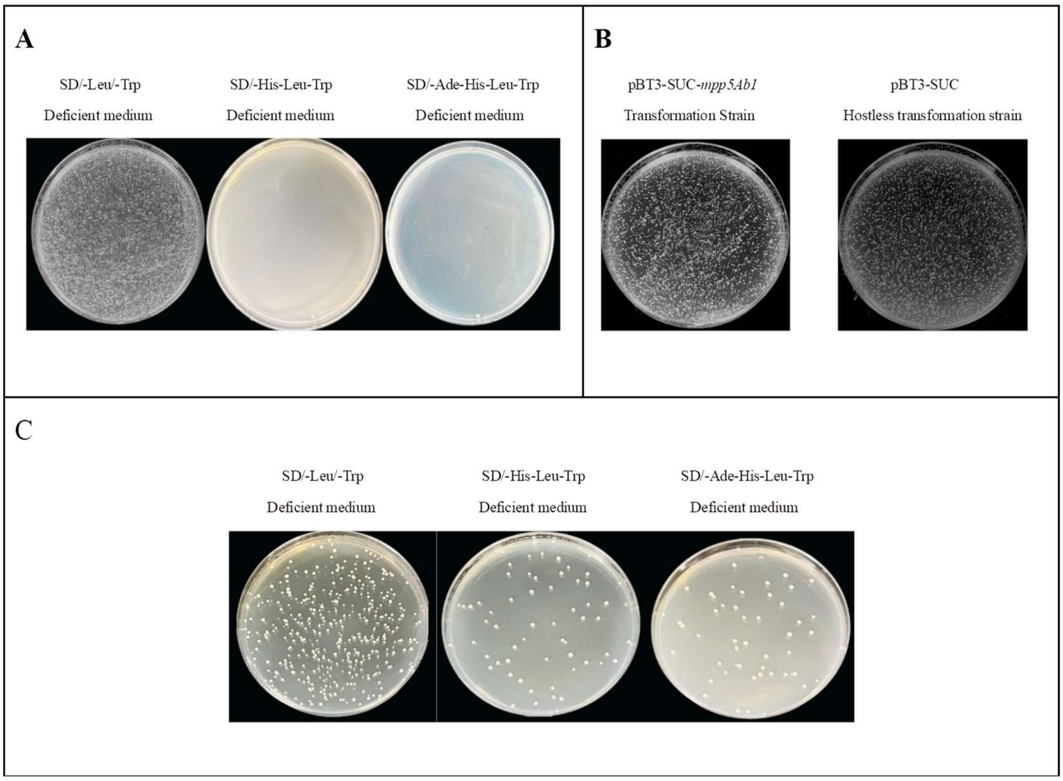

Figure S5. A, Self-activation test of the bait plasmid. B, Toxicity Assessment of Recombinant Plasmid. C, Functional verification test of the bait plasmid.

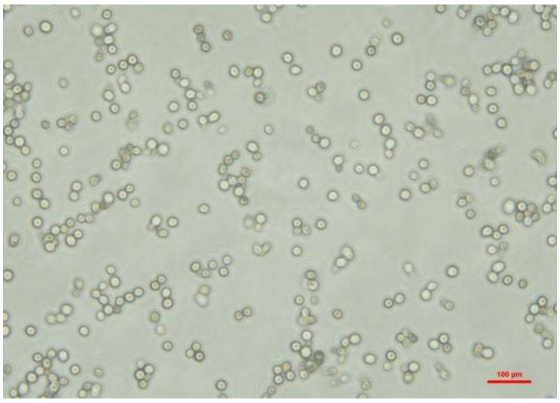

Figure S6. Microscopic Examination of Hybridization Solutio.

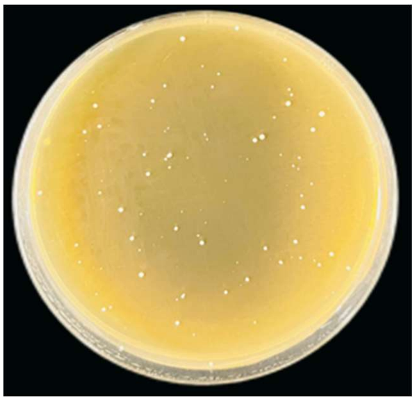

Figure S7. Colonies on QDO plate.

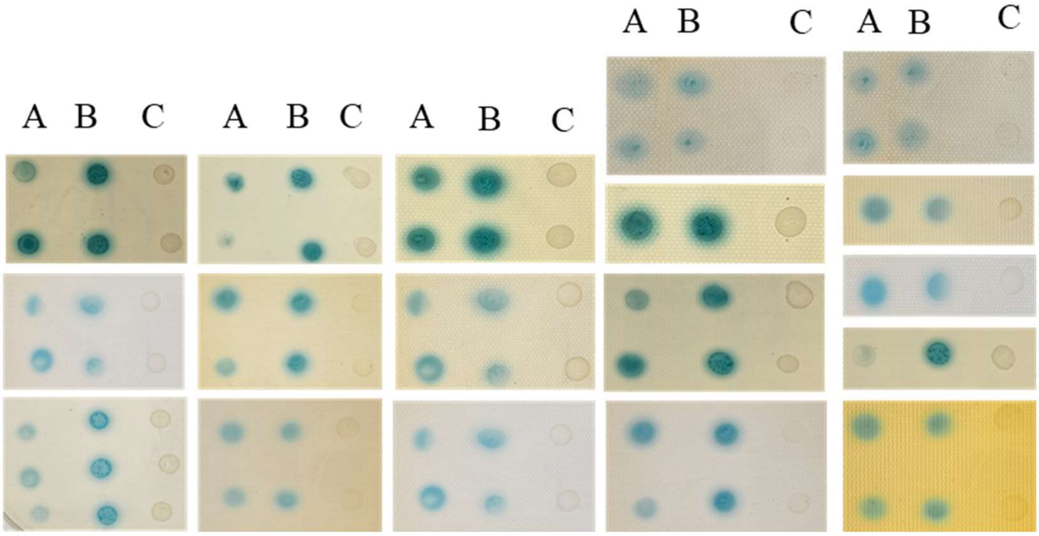

Figure S8. Screening resμLts of interacting proteins.

Note: A: Total positive clones transformed, B: Positive control, C: Negative control

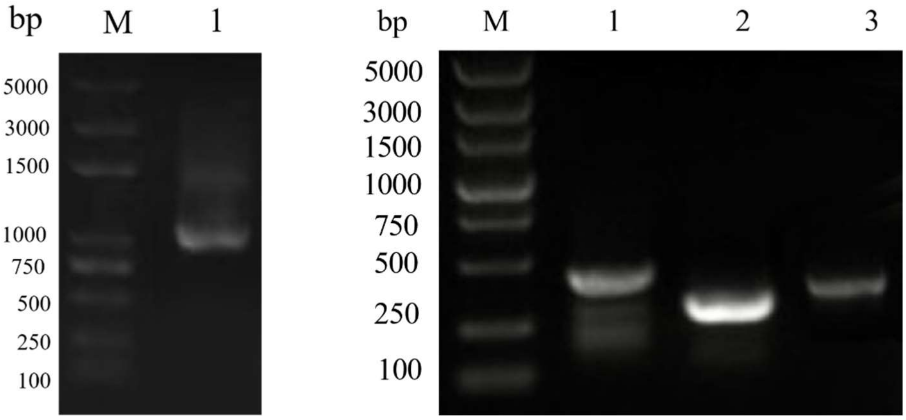

Figure S9. Colony PCR Verification of BiFC Plasmid. M: DL5000 DNA marker, 1-3: Reconstituted coloniesPCR.
